# Transcriptomic Profiling Reveals Potential Genes Involved in the Immune Landscape of Polycystic Ovary Syndrome: An Exploratory Study

**DOI:** 10.1007/s43032-025-01917-4

**Published:** 2025-06-24

**Authors:** Ye Zhang, Zhiyang Hu, Zebo Cai, Junda Lai, Huiqiong Zeng

**Affiliations:** 1 Department of Immunology, Traditional Chinese Medicine, Women & Children Health Institute Futian Shenzhen, Shenzhen, 518000 China; 2 Department of Obstetrics, Shenzhen People’s Hospital, The Second Clinical Medical College, Jinan University, The First Affiliated Hospital, Southern University of Science and Technology, Shenzhen, 518000 China; 3 Department of Human Life Sciences, Beijing Sport University, Haidian District, #48 Xinxi Road, Beijing, 100029 China

**Keywords:** Polycystic ovary syndrome, Anoikis, Machine learning, Diagnostic biomarkers, Drug prediction, Immune landscape, Pancancer

## Abstract

**Background:**

Polycystic ovary syndrome (PCOS) is a complex endocrine disorder associated with chronic inflammation, insulin resistance, and ovarian dysfunction. Emerging evidence implicates granulosa cell anoikis, a specialized form of apoptosis induced by extracellular matrix detachment, in PCOS pathogenesis. However, the involvement of anoikis-related genes (ARGs) remains poorly understood.

**Methods:**

Transcriptomic data from two GEO datasets (GSE43264 and GSE98421) were integrated, followed by differential gene expression analysis and weighted gene co-expression network analysis (WGCNA). Thirteen differentially expressed ARGs (DEARGs) were identified. Functional enrichment (GO/KEGG), immune infiltration profiling (CIBERSORT), and machine learning models were used to screen for hub biomarkers. Diagnostic potential was validated using ROC and nomogram analyses. ceRNA, drug-gene, and transcription factor networks were constructed. Pancancer expression and immune relevance were assessed using TCGA, and gene function was validated through MGI.

**Results:**

Two ARGs, GSTP1 and LPCAT1, were identified as robust diagnostic markers (AUC > 0.80). These genes were significantly associated with immunosuppressive cell infiltration (e.g., elevated M2 macrophages, reduced CD8⁺ T cells). GSEA linked both genes to apoptosis, PI3K signaling, and immune pathways. MGI validation revealed that *GSTP1* and *LPCAT1* are involved in reproductive and metabolic regulation in murine models, supporting their functional relevance to PCOS. Drug prediction analyses identified resveratrol, curcumin, and vitamin E as potential therapeutics.

**Conclusion:**

This is the first exploratory study to identify *GSTP1* and *LPCAT1* as potential diagnostic biomarkers for PCOS, validated across multi-omics platforms and functional databases. These findings highlight a novel anoikis-immunity axis in PCOS and suggest new directions for biomarker-guided therapy.

**Supplementary Information:**

The online version contains supplementary material available at 10.1007/s43032-025-01917-4.

## Introduction

Polycystic ovary syndrome (PCOS) is a highly prevalent and multifaceted endocrine disorder, impacting an estimated 10–15% of women during their reproductive years [1]. Despite its high prevalence and association with infertility, insulin resistance, and low-grade inflammation, the fundamental molecular mechanisms underlying PCOS remain elusive [2]. Current clinical management focuses on alleviating symptoms through the use of combined oral contraceptives, insulin-sensitizing agents like metformin, and assisted reproductive technologies [3]. Immune dysregulation in PCOS involves chronic low-grade inflammation, altered macrophage and T-cell activity, and disrupted cytokine balance. Imbalances in T helper 17 cells / Regulatory T cells (Th17/Treg) ratios and increased auto-antibodies contribute to ovarian dysfunction, insulin resistance, and infertility [4]. However, the fundamental pathogenic mechanisms that drive PCOS remain incompletely understood.

Granulosa cells (GCs), which surround the developing oocyte, play essential roles in folliculogenesis, and their survival is critical for ovulatory competence. Recent studies suggest that excessive apoptosis and adhesion instability of GCs contribute to follicular arrest in PCOS [5]. Nevertheless, while apoptosis in GCs is a recognized feature of PCOS, the precise form of cell death, particularly anoikis under conditions of extracellular matrix (ECM) disruption, is less understood. Anoikis is a specific form of programmed apoptosis that is triggered by the loss of cell-ECM interactions [6]. Originally identified in epithelial systems, anoikis acts as a safeguard against ectopic survival of dislocated cells and is tightly regulated by integrins, focal adhesion kinase (FAK), and downstream pro-apoptotic factors such as BIM and Bad [7]. Although anoikis has been extensively studied in cancer biology and tissue regeneration, its relevance to reproductive endocrinology remains largely unexplored [8, 9]. Notably, transcriptomic and proteomic analyses of PCOS ovarian tissue and follicular fluid suggest aberrations in ECM structure and focal adhesion dynamics, and integrin signaling, potentially revealing an adhesion-deficient microenvironment [10, 11]. Changes in the ECM and cellular death mechanisms are observed in PCOS [12]. Moreover, Han et al. discloses that Programmed Death-Ligand 1 (PD-L1) enhances GCs survival via the phosphoinositide 3-kinase/protein kinase B (PI3K/AKT) signaling pathway and regulates ECM-related factors such as Collagen Type I Alpha 1 Chain (COL1A1) [13]. A study reveals that growth hormone (GH) alleviates oxidative stress (OS)-induced apoptosis in PCOS GCs by inhibiting mitochondrial dysfunction, caspase-9 activation, and Bad phosphorylation via the PI3K/Akt-FOXO1 pathway [14]. These findings raise the possibility that anoikis resistance may underlie follicular persistence in PCOS. Despite this theoretical framework, no study has yet comprehensively characterized anoikis-related genes (ARGs) in PCOS. To address this gap, we constructed a predictive model to evaluate the prognostic value of ARGs in PCOS. This study represents the first systematic characterization of ARG expression patterns in PCOS, aiming to elucidate their biological and immunological functions, with further exploration of their roles in the immune microenvironment and across pan-cancer contexts.

## Materials and Procedures

### Data Selection

**Fig. 1:**
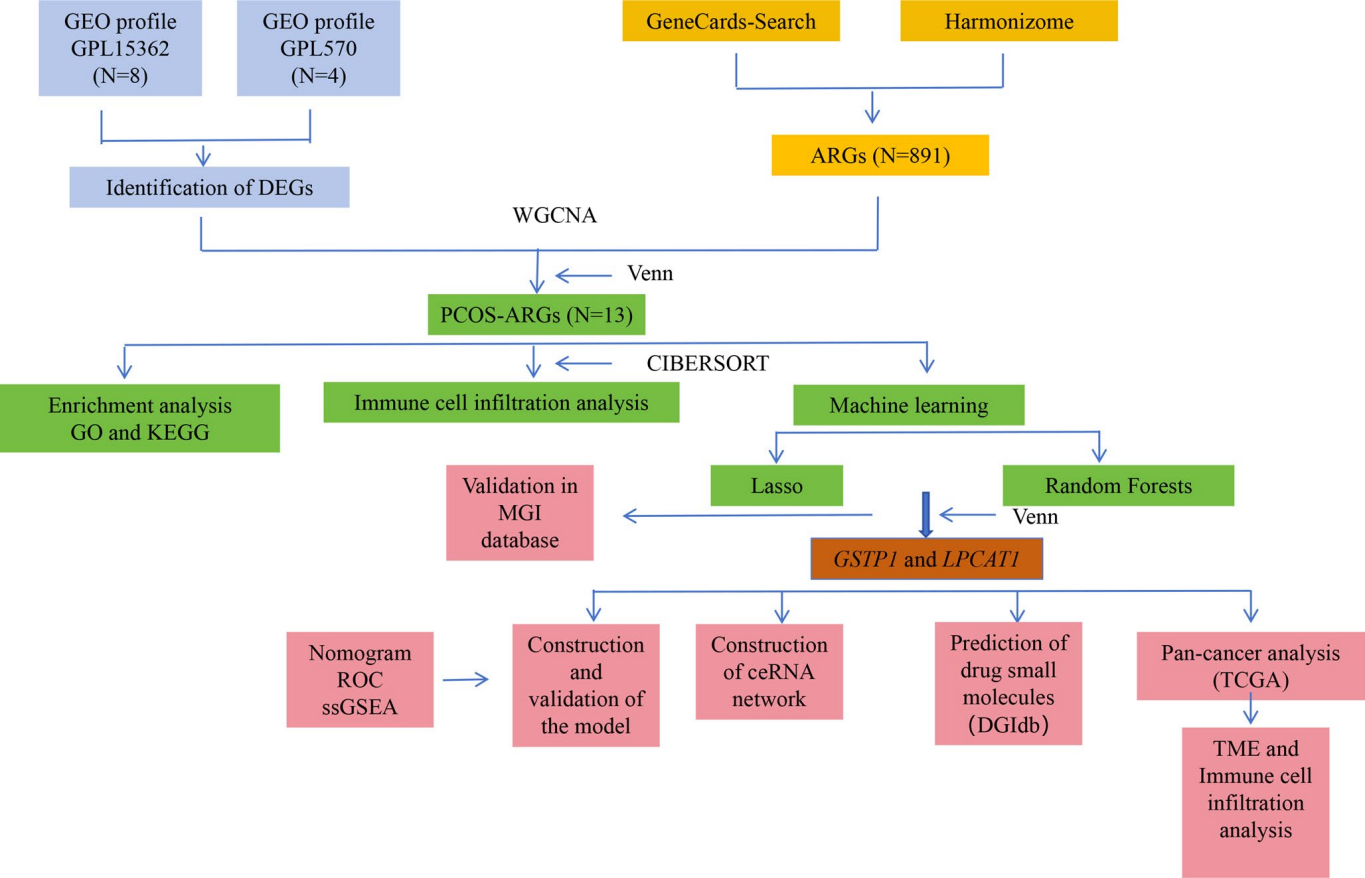
Study flow. Abbreviations: GEO (Gene Expression Omnibus), PCOS (Polycystic Ovary Syndrome), DEGs (Differential Expression Genes), WGCNA (Weighted Gene Co-expression Network Analysis), ARGs (Anoikis-Related Genes), MGI (Mouse Genome Informatics) database, GO (Gene Ontology), KEGG (Kyoto Encyclopedia of Genes and Genomes), ROC (Receiver Operating Characteristic), GSEA (Gene Set Enrichment Analysis), LASSO (Least Absolute Shrinkage and Selection Operator), TCGA (The Cancer Genome Atlas); TME (Tumor Microenvironment)

Two original datasets of PCOS patients (GSE43264 and GSE98421) were obtained from the publicly available GEO database. The GSE43264 dataset (annotated platform: GPL15362) included 8 PCOS samples and 7 healthy control (HC) samples (https://www.ncbi.nlm.nih.gov/geo/query/acc.cgiacc=GSE43264), while the GSE98421 dataset (annotated platform: GPL570) comprised 4 PCOS samples and 4 HC samples (https://www.ncbi.nlm.nih.gov/geo/query/acc.cgiacc=GSE98421). Datasets were selected based on the availability of raw gene expression data from human PCOS ovarian tissue or GCs, clear distinction between PCOS cases and healthy controls, and adequate sample size for differential gene expression analysis. The expression profiles of these datasets were integrated to form a test set, with the SVA algorithm used to correct for interchip data. Principal component analysis (PCA) was applied to evaluate the performance of the comBat algorithm [15]. The probe ID of each gene was translated into a gene symbol (Table 1). If a gene symbol corresponded to multiple probe IDs, the average expression value of the probe IDs was calculated as the representative expression value of the gene. Information on 891 ARGs was collected from GeneCards-Search (www.genecards.org/) and Harmonizome (https://maayanlab.cloud/Har). The study flow chart is shown in Fig. 1.

**Table 1:** Data source

GEO dataset	NPCOS	NCONTROL	annotated platform	Expression profiling
GSE43264	8	7	GPL15362	array
GSE98421	4	4	GPL570	array

### Screening Differentially Expressed Genes Associated with PCOS

The processing and statistical analysis of the expression files were performed using *R* software (version 4.1.2) [16]. The “limma” package in *R* was used for normalizing the data and identifying differentially expressed genes (DEGs), with significant DEGs defined as those whose log fold change (logFC) was greater than 1.2 and whose *P* value was less than 0.05. The “ggplot2” package was used to plot the volcano plot of the DEGs to visualize their distribution. Moreover, with the assistance of the “pheatmap” *R* package, the expression data of DEGs are represented as a heatmap for a more intuitive display.

### WGCNA

Through the implementation of weighted gene coexpression network analysis (WGCNA), we sought to identify synergistically expressed gene modules and investigate the associations between gene networks and phenotypes, as well as the hub genes within the network [17]. We utilized the WGCNA-*R* package to construct the coexpression network of all genes in the dataset to facilitate further analysis, with a soft-thresholding power of 19. Subsequently, the weighted adjacency matrix was transformed into a topological overlap matrix (TOM) to estimate network connectivity, with hierarchical clustering methods employed to construct the dendrogram of the TOM matrix [18]. Each branch of the dendrogram represents a different gene module, denoted by a distinct color. Based on the weighted correlation coefficient of the genes, they were classified according to their expression patterns, with genes of similar patterns grouped into a module. Consequently, tens of thousands of genes were segregated into multiple modules based on gene expression patterns. Using the online Venn tool, the intersection of differentially expressed genes (DEGs), key module genes from WGCNA, and ARGs were identified, ultimately revealing 13 hub ARGs [19].

### Enrichment Analysis of Advanced Functions

Gene Ontology (GO) is a widely utilized database in bioinformatics, encompassing approximately 7 million gene annotations, of which approximately 10% have been experimentally validated [20]. GO analysis was employed to examine cellular components (CCs), biological processes (BPs), and molecular functions (MFs) associated with the candidate genes identified in this study. The Kyoto Encyclopedia of Genes and Genomes (KEGG) is another database intended for the analysis of complex functions and biological systems and for constructing pathway maps based on gene sequences, genomic information, metabolism, diseases, and other relational networks [21]. To evaluate the enrichment of hub ARG functions and pathways, we applied the “clusterProfiler” package to perform enrichment analysis on 13 ARGs [22] and the “org.db” and “ggplot2” packages for data visualization [23, 24]. Only when the *P* value was less than 0.05 were the GO and KEGG enrichment pathways considered to be significantly enriched.

### Immune Cell Infiltration Analysis

CIBERSORT is a technique that leverages expression data to map the cellular composition of complex tissues by quantitatively measuring the abundance of 22 types of tumor-infiltrating immune cells in samples [25]. Applying this algorithm to RNA-seq data from PCOS patients revealed the relative proportions of these 22 types of infiltrating immune cells. The “forcats” and “tidyHeatmap” packages were subsequently used to visualize the estimated compositional proportions of immune cell subtypes within each sample. The “corrplot” package was used to investigate the interaction dynamics among immune cells, further elucidating the interplay among tumor immune cells. The “vioplot” package presents the disparity in the relative content of immune cells between the PCOS and control groups. A *P* value less than 0.05 indicated a significant difference. Finally, Spearman correlation was utilized to examine the relationships between key regulatory factors, immune cells, and the infiltration levels of the hub ARGs.

### Selection of Characteristic Genes in Machine Learning

Machine learning (ML), as an implementation of artificial intelligence (AI), is capable of autonomously analyzing and interpreting data to extract rules or models and uses these data as guidelines to predict unknown data [26]. We employed two ML techniques for in-depth filtration of potential diagnostic PCOS genes. The Least Absolute Shrinkage and Selection Operator (LASSO) regression [27] is a regularization technique that improves prediction accuracy and model interpretability by shrinking less relevant coefficients to zero, thereby enabling effective feature selection. This is particularly advantageous in high-dimensional settings such as genomics, where the number of variables far exceeds the number of samples. Random Forest (RF) [28], a non-parametric ensemble learning method, imposes no strict assumptions on variable distributions and is robust to noise, non-linearity, and outliers. It offers high accuracy, sensitivity, and specificity, while also providing measures of variable importance, making it valuable for both prediction and biological insight. We implemented LASSO regression and RF analysis using the “glmnet” and “randomForest” packages in *R*. The genes selected by both methods are considered hub ARGs in the diagnosis of PCOS. These methods were selected due to their complementary strengths in addressing the challenges of high-dimensional gene expression data and providing robust feature selection [29]. The nomogram exhibits stability and precision, rendering it applicable to clinical patient management [30]. It is widely used for risk assessment, prognosis evaluation, and treatment decision-making.

We conducted a nomogram analysis to predict the recurrence of PCOS using the “Rms” program. Nomogram analysis elucidated the impact of each predictive ARG on PCOS. The model evaluation encompasses calibration plots to assess accuracy, receiver operating characteristic (ROC) curves for model performance, and decision curve analysis (DCA) for evaluating clinical utility. The calibration curve was used to compare the predicted and actual results. Matching the diagonal line showed identical outcomes. The curve fit indicated accurate prediction (on the line) or overestimation (below the line) of risks. We utilized the “pROC” package in *R* for ROC analysis to further investigate the precision of our model’s predictions [31]. All analyses were performed using *R* software (version 4.1.2).

### Gene Set Enrichment Analysis

GSEA aims to elucidate the biological significance of characteristic genes from a functional perspective. We utilized the gene set “c2.cp.kegg.v7.4.symbols.gmt” from the Molecular Signatures Database (MSigDB, http://software.broadinstitute.org/gsea/msigdb) as the reference set. To acquire the normalized enrichment score for each analysis, a permutation of the gene set was conducted once [32]. Enrichment was considered significant when the false discovery rate (FDR) was less than 0.05.

### Establishing a ceRNA Network

The multiMiR *R* package, a tool for predicting miRNA binding sites, was used to identify miRNAs that may interact with DEGs. Next, an intersection analysis of miRNA-DEGs was performed to establish a ceRNA network of mRNAs [33]. The results were visualized using Cytoscape 3.8.0 software [34]. To delve into the molecular mechanisms of the hub genes, the NetworkAnalyst database (www.networkanalyst.ca/) was used to identify TF-target gene interactions, creating a regulatory network between transcription factors and target genes [35]. The drug–gene interaction data were obtained from the Drug–Gene Interaction database (DGIdb, www.dgidb.or) and then imported into Cytoscape for further visualization [36]. This analysis was intended to explore potential compounds that may interact with the identified ARGs in a hypothesis-generating manner.

### Pancancer Analysis of the Hub Genes

We retrieved comprehensive cancer mRNA expression data of 33 types of tumors associated with pancancer from The Cancer Genome Atlas (TCGA) database (https://portal.gdc.cancer.gov/projects/TCGA) and clinical information, thereby further investigating the association between gene expression and subsequent analysis [37]. Survival analysis for each cancer type (*P* < 0.05) was conducted using the Kaplan‒Meier method, with the “survival” and “survminer” packages utilized for assessment of overall survival (OS) and progression-free interval (PFI) [38]. Additionally, we employed the “survival” and “forestplot” packages for Cox regression analysis, evaluating the correlation between ARG expression and survival across cancer types, with the broader aim of uncovering conserved or divergent gene-behavior relationships applicable to systemic disease frameworks.

### Assessing the Tumor Microenvironment

Estimation of STromal and Immune cells in MAlignant Tumor tissues using Expression data (ESTIMATE) is a computational method based on gene expression to evaluate immunocyte infiltration and the tumor microenvironment [39]. It is capable of calculating immune scores and stromal scores and estimating scores among samples. We utilized the CIBERSORT algorithm to analyze RNA-seq data from patients with 33 different cancer subtypes, with the aim of inferring the relative proportions of infiltrating immunocytes and carrying out correlation analysis on gene expression and the content of immunocytes. In addition, potential relationships between gene expression and immune regulatory factors were explored via the TISIDB website (http://cis.hku.hk/TISIDB/) [40]. TISIDB is a specialized database dedicated to the study of interactions between tumors and the immune system. It compiles a vast amount of data related to tumor immunology.

### Validation in Mouse Genome Informatics Database

We utilized the Mouse Genome Informatics (MGI) database (http://www.informatics.jax.org/) to validate candidate proteins identified through prior statistical analyses. Our investigation centered on two experimentally curated MGI modules: “Mutations, Alleles, and Phenotypes” and “Expression”, which bridge functional genomics insights with translational relevance [41]. The MGI database supports functional validation of candidate genes using knock-out mouse models. By integrating loss-of-function phenotypes and tissue-specific expression data, MGI helps prioritize genes relevant to PCOS’ mechanisms, particularly in reproductive and immune systems, and facilitates hypothesis generation on gene function and pathogenesis.

### Statistical Analysis

All the statistical analyses were carried out using *R* software (version 4.1.2). The degree to which the model predicted outcomes was measured using ROC curve analysis. Differences in immune infiltrating cells were compared using the Wilcoxon test. Visualization was conducted using the “ggplot2”, “pheatmap”, and “forestplot” packages. A *P* value of less than 0.05 was considered to indicate statistical significance. The Wilcoxon test was used to analyze differences between the two groups. All the statistical tests were two-sided, and values of *P* < 0.05 were considered to indicate statistical significance.

## Results

### Identification of Differentially Expressed Genes in PCOS

**Fig. 2:**
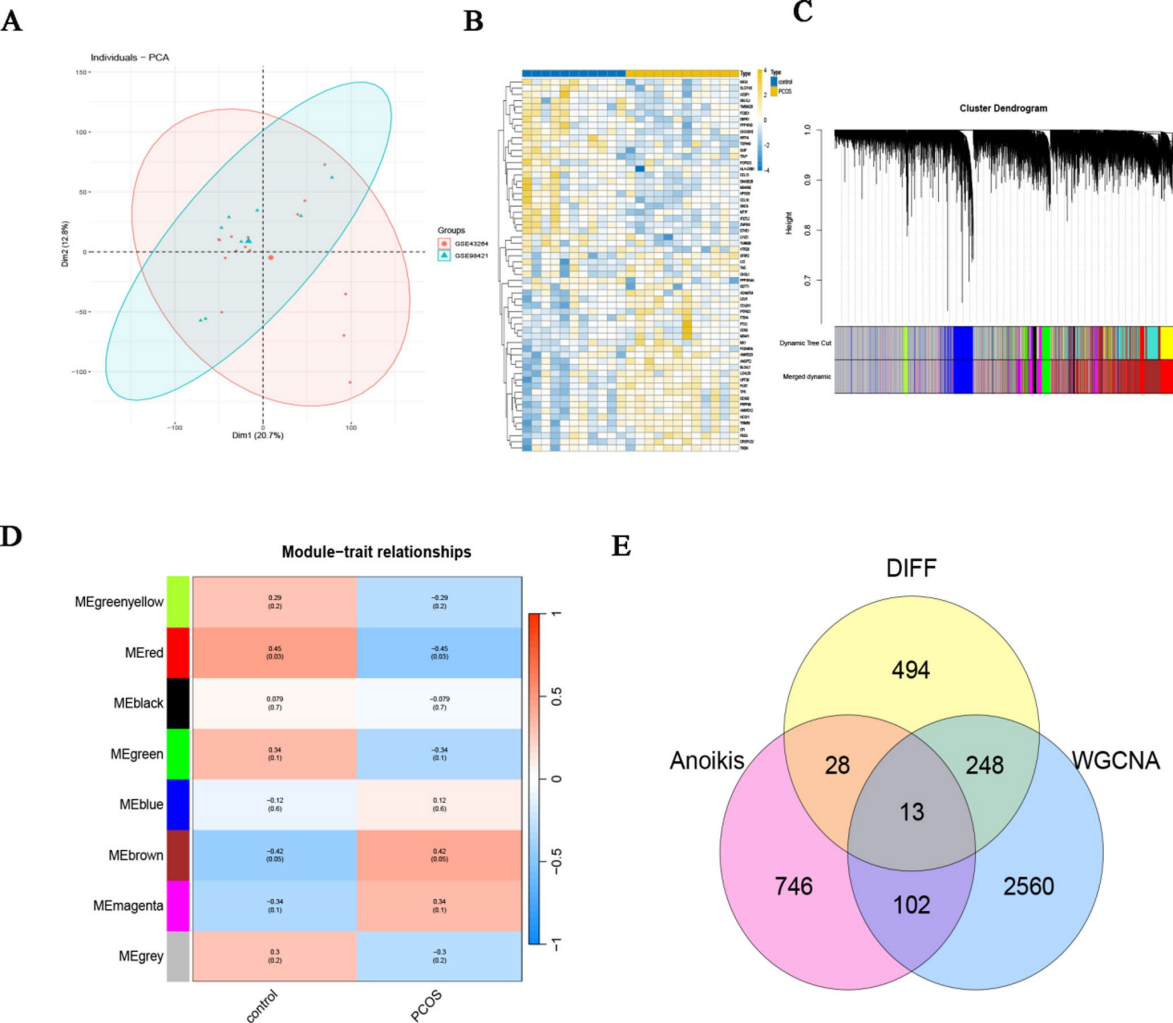
Identification of anoikis-related differentially expressed genes (DEGs) and co-expression modules. (**A**) Principal component analysis (PCA) showing the sample distribution and batch effect between the two datasets (GSE43264 and GSE98421). Each dot represents a sample; colors indicate dataset origin. (**B**) Heatmap of differentially expressed genes (DEGs) in polycystic ovary syndrome (PCOS) samples compared to controls. Yellow and blue represent high and low expression levels, respectively. (**C**) Cluster dendrogram of genes based on weighted gene co-expression network analysis (WGCNA). Each branch represents a gene, and color bars below indicate module membership. (**D**) Module-trait relationships between gene modules and PCOS/control groups. The color scale reflects correlation coefficients, with red indicating positive and blue indicating negative correlations. Each cell shows the correlation coefficient and p-value in parentheses. (**E**) Venn diagram showing overlap among DEGs (DIFF), anoikis-related genes (ARGs), and key module genes identified by WGCNA. The intersection of the three sets indicates potential ARGs associated with PCOS

We acquired two datasets (GSE43264 and GSE98421) from the GEO online database, which included 11 control subjects and 12 PCOS patients, as mentioned above. Before batch correction, the uniform manifold approximation and projection (UMAP) outcomes of the two datasets, differentiated by color, were separated and independent of each other (Figure S1). After eliminating interbatch differences, the sample distribution between datasets tended to be consistent (Fig. 2A). Subsequently, we conducted differential analysis on the control group and PCOS group, identifying 783 differentially expressed genes (DEGs), including 545 upregulated genes and 238 downregulated genes. The DEGs were visualized using heatmaps (Fig. 2B). The top five upregulated genes were *BAG1*,* ALDH8A1*,* HEY1*,* SNRK*, and *C16orf52*, while the top five downregulated genes were *ZFP42*,* ALKBH6*,* ACAN*,* CST4*, and *PRSS12*.

### Identifying Key Gene Modules in PCOS Via WGCNA

To deepen our understanding of the underlying mechanisms and gene modules in PCOS, we conducted batch normalization of disease samples in the dataset. A soft-thresholding power of 19 was selected when *R*^*2*^ was greater than 0.9 and the average connectivity was high. After strongly correlated modules were merged using a height cutoff of 0.25 in the dendrogram, the initial and merged modules were finally visualized under the clustering tree (Fig. 2C). Intramodular connectivity analysis confirmed the reliability of the module description, demonstrating no substantial linkages between modules (Figure S2). The positive correlation between module eigengene (ME) values and clinical features was used to investigate the association between the modules and clinical symptoms. The brown module displayed the strongest positive correlation with PCOS, identifying a meaningful key module (Fig. 2D). The brown module contains 2923 genes, which showed the highest correlation with PCOS-related genes among all the modules, with an absolute Pearson correlation coefficient (*r*) of 0.32. The results showed a strong correlation between the brown module and PCOS in the module membership (MM) versus gene significance (GS) scatter plot for PCOS (Figure S3). By comparing DEGs with the WGCNA key module genes and anoikis intersection using Venn online tools, we identified 13 differentially expressed anoikis-related genes (DEARGs), namely, *ANKRD13C*,* PIK3R1*,* ITGA4*,* PRPF4B*,* HK2*,* GSTP1*,* XAF1*,* OGT*,* SNCG*,* LPCAT1*,* EDNRB*,* ICAM1*,* and SS18* (Fig. 2E).

### Enrichment Analysis of DEARGs

**Fig. 3:**
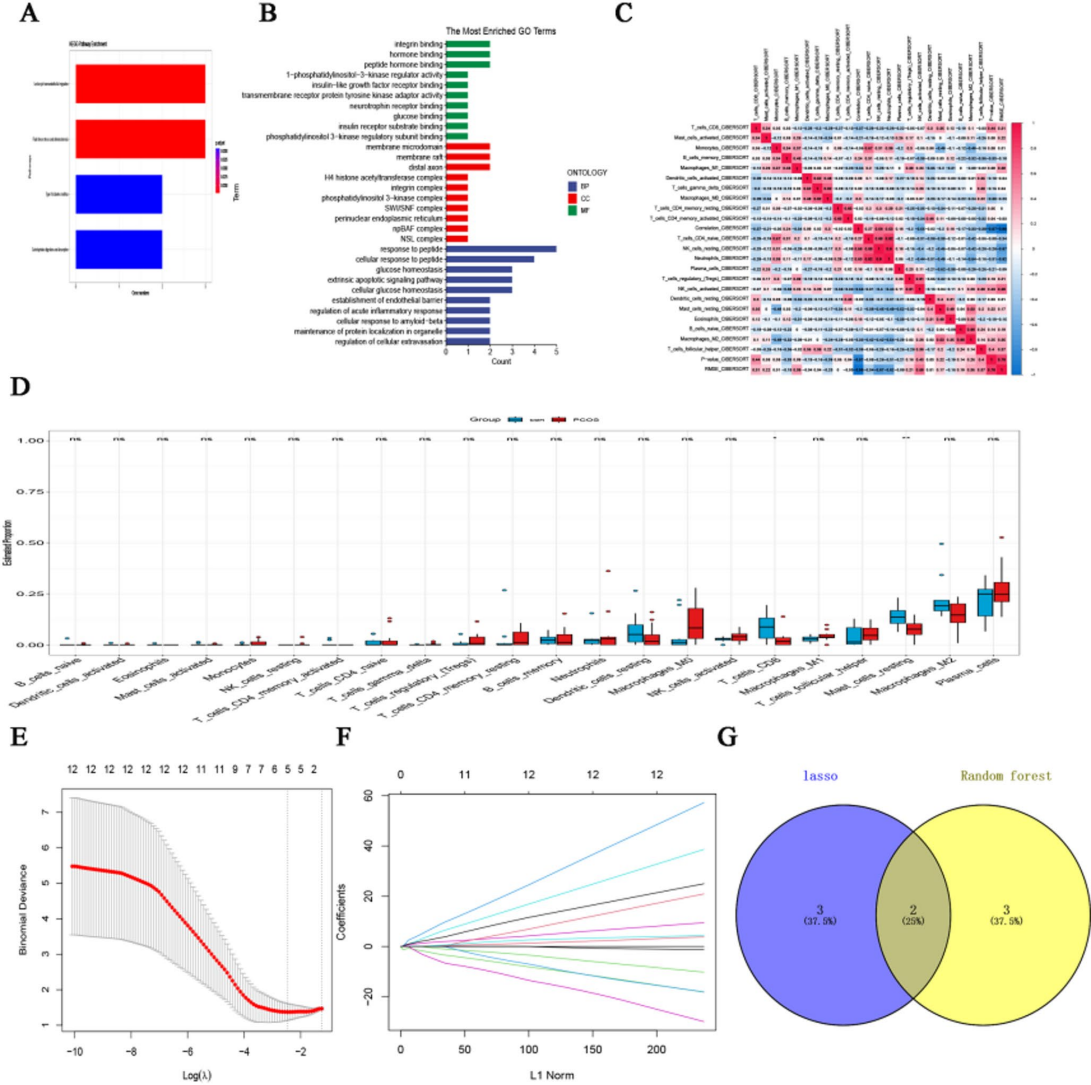
Functional enrichment, immune infiltration analysis, and machine learning screening of ARGs in PCOS. (**A**) Kyoto Encyclopedia of Genes and Genomes (KEGG) pathway enrichment analysis of PCOS-related ARGs, showing the top enriched signaling pathways. (**B**) Gene Ontology (GO) enrichment analysis of PCOS-related ARGs, classified into biological process (BP), cellular component (CC), and molecular function (MF) categories. (**C**) Heatmap showing pairwise correlations among immune cell types in PCOS samples. Red and blue colors indicate positive and negative correlations, respectively; numeric values represent correlation coefficients. (**D**) Boxplots comparing the relative abundance of immune cell types between PCOS and control groups. Boxes show interquartile ranges, and whiskers indicate data variability. Statistical significance: *P* < 0.05 (*), *P* < 0.01 (**), ns = not significant. (**E**) LASSO logistic regression model used for feature selection of ARGs. The plot shows tenfold cross-validation results across different log(λ) values; the vertical dotted line marks the optimal λ with the minimum binomial deviance. (**F**) LASSO coefficient profiles of ARGs along the L1 regularization path. Five genes with non-zero coefficients were selected for further analysis. (**G**) Venn diagram showing the overlapping ARGs identified by both LASSO and Random Forest algorithms. The intersection indicates the common hub genes selected by both machine learning methods. Percentages represent the proportion of genes in each segment

A detailed analysis was conducted on the differential DEARGs via both GO and KEGG pathway analyses. According to the KEGG enrichment analysis, notable gene-enriched pathways predominantly included leukocyte transendothelial migration, fluid shear stress and atherosclerosis, among others (Fig. 3A). According to the GO-MF analysis, the primary processes involved reactions to 1-phosphatidylinositol-3-kinase regulator activity (GO:0046935) and insulin-like growth factor receptor binding (GO:0005159). GO-CC revealed membrane microdomains (GO:0098857) and phosphatidylinositol 3-kinase complex (GO:0005942) as the main pathways. Finally, in the GO-BP analysis, the dominant pathways included integrin binding and hormone binding (GO:0005178) (Fig. 3B).

### Evaluation of Immune Cell Infiltration

Using the CIBERSORT algorithm, we identified 22 types of immune cells and constructed a box plot to visualize the relative abundances of these immune cell infiltration subgroups. We utilized the “corrplot” package to investigate the interaction relationships between immune cells, facilitating a deeper analysis of the influence of immune cell interaction relationships on disease states (Fig. 3C). In addition, we employed the “ggpubr” package to graphically depict the variations in relative immune cell abundance among the different groups (Fig. 3D).

Compared to the control group, PCOS patients exhibited significant decreases in the numbers of resting mast cells and CD8⁺ T cells. In addition, CIBERSORT analysis revealed lower infiltration of memory B cells and plasma cells, while levels of M2 macrophages, regulatory T cells (Tregs), and γδ T cells were relatively elevated.

### Machine Learning-Based Identification of Hub Genes

We utilized two machine learning (ML) algorithms to identify signature genes. LASSO logistic regression analysis, based on the least squares method, was applied to fit the expression profiles of the ARGs. With this approach, we determined the optimal value for λ and selected five predictive ARGs (as shown in Fig. 3E and F). Additionally, we integrated the RF algorithm with feature selection to determine the error rate and the number of decision trees (as shown in Figure S4). By cross-referencing both methods, we used a Venn diagram to successfully identify two overlapping genes, *GSTP1* and *LPCAT1* (as shown in Fig. 3G).

### Construction and Validation of a Prognostic Feature Model for Hub-Associated ARGs

**Fig. 4:**
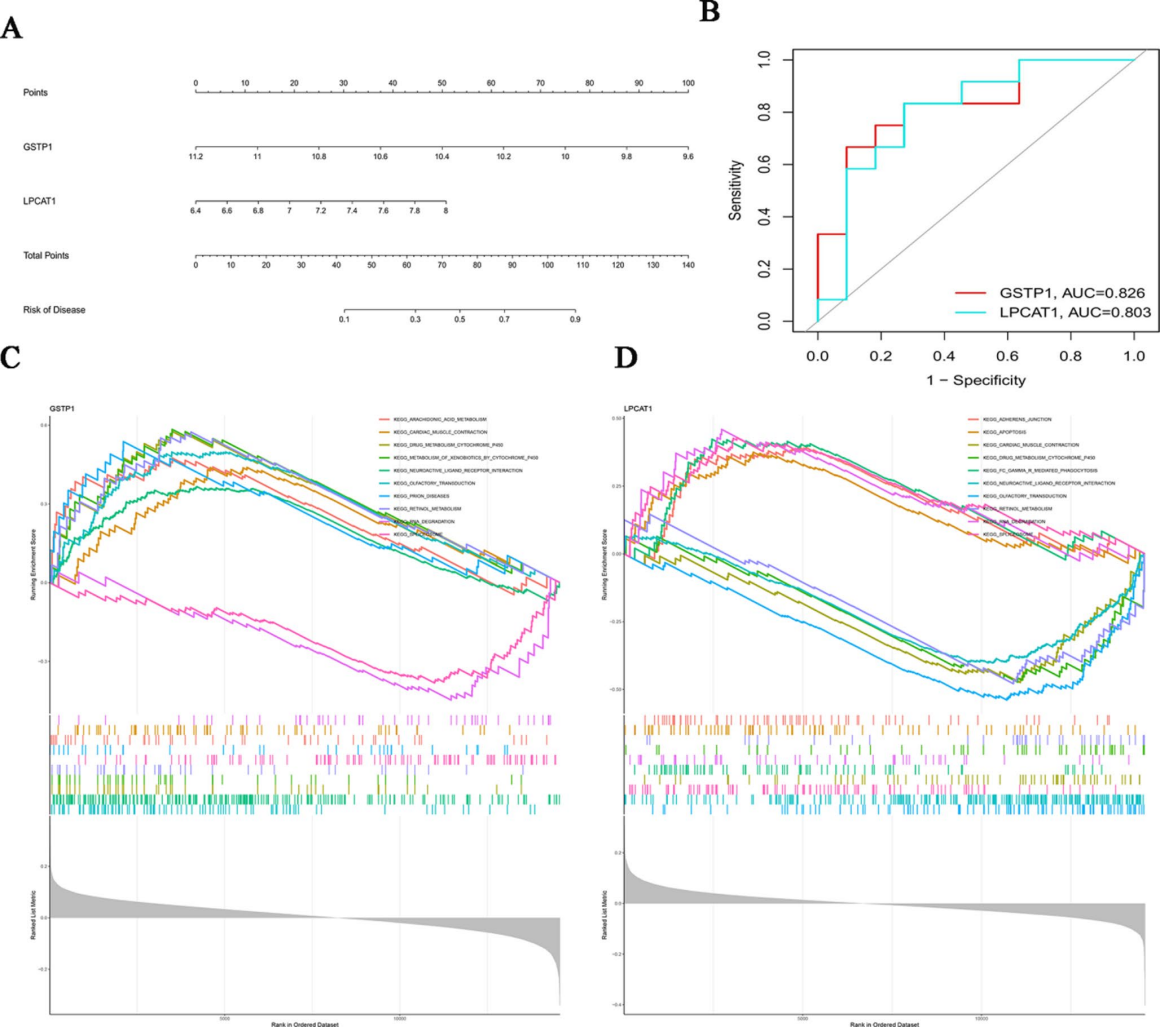
Construction and validation of a predictive model based on hub ARGs in PCOS. (**A**) A nomogram model integrating the expression levels of *GSTP1* and *LPCAT1* to predict disease risk in PCOS patients. Points are assigned to each gene and summed to estimate the total risk score. (**B**) Receiver operating characteristic (ROC) curves evaluating the diagnostic performance of GSTP1 (AUC = 0.826) and LPCAT1 (AUC = 0.803) in distinguishing PCOS from control samples. (**C**) Single-sample gene set enrichment analysis (ssGSEA) of *GSTP1*, showing its association with multiple KEGG pathways. The plot ranks samples by expression level and highlights pathway enrichment scores. (**D**) ssGSEA results for *LPCAT1*, displaying pathway-level enrichment patterns across samples. Color-coded lines indicate different KEGG functional categories

We utilized the Rms package (Fig. 4A) to develop a nomogram model for the diagnosis of PCOS based on selected hub ARGs (*GSTP1* and *LPCAT1*) and assessed its predictive power through calibration curves. The calibration curve demonstrated a minimal difference between the actual PCOS risk and the predicted PCOS risk, suggesting the high accuracy of the nomogram model for PCOS diagnosis (Figure S5). The accuracy of the risk model was further confirmed through ROC curve analysis (AUC = 0.841, 95% CI: 0.659 − 0.970). In the decision curve analysis (DCA), the “nomogram” curve was above the gray line, indicating that patients can benefit from the nomogram risk score model under a high-risk threshold of 0 to 1 (Figure S6). To further validate the diagnostic value of *GSTP1* and *LPCAT1*, we performed ROC analysis on two genes: *GSTP1* (AUC: 0.826) and *LPCAT1* (AUC = 0.803) (Fig. 4B). These findings imply a significant association between all the main ARGs and PCOS. We conducted GSEA on the two hub ARGs to gain deeper insights into biological processes and to predict potential signaling pathways in which hub ARGs are expressed in PCOS patients. The results showed that *GSTP1* was mainly enriched in the KEGG-ARACHIDONIC ACID METABOLISM (*P* = 1.64E-07) and KEGG-APOPTOSIS (*P* = 0.011) pathways (Fig. 4C); *LPCAT1* was primarily enriched in the KEGG-T CELL RECEPTOR SIGNALING PATHWAY (*P* = 0.037) and KEGG-APOPTOSIS (*P* = 0.005) pathways (Fig. 4D).

**Fig. 5:**
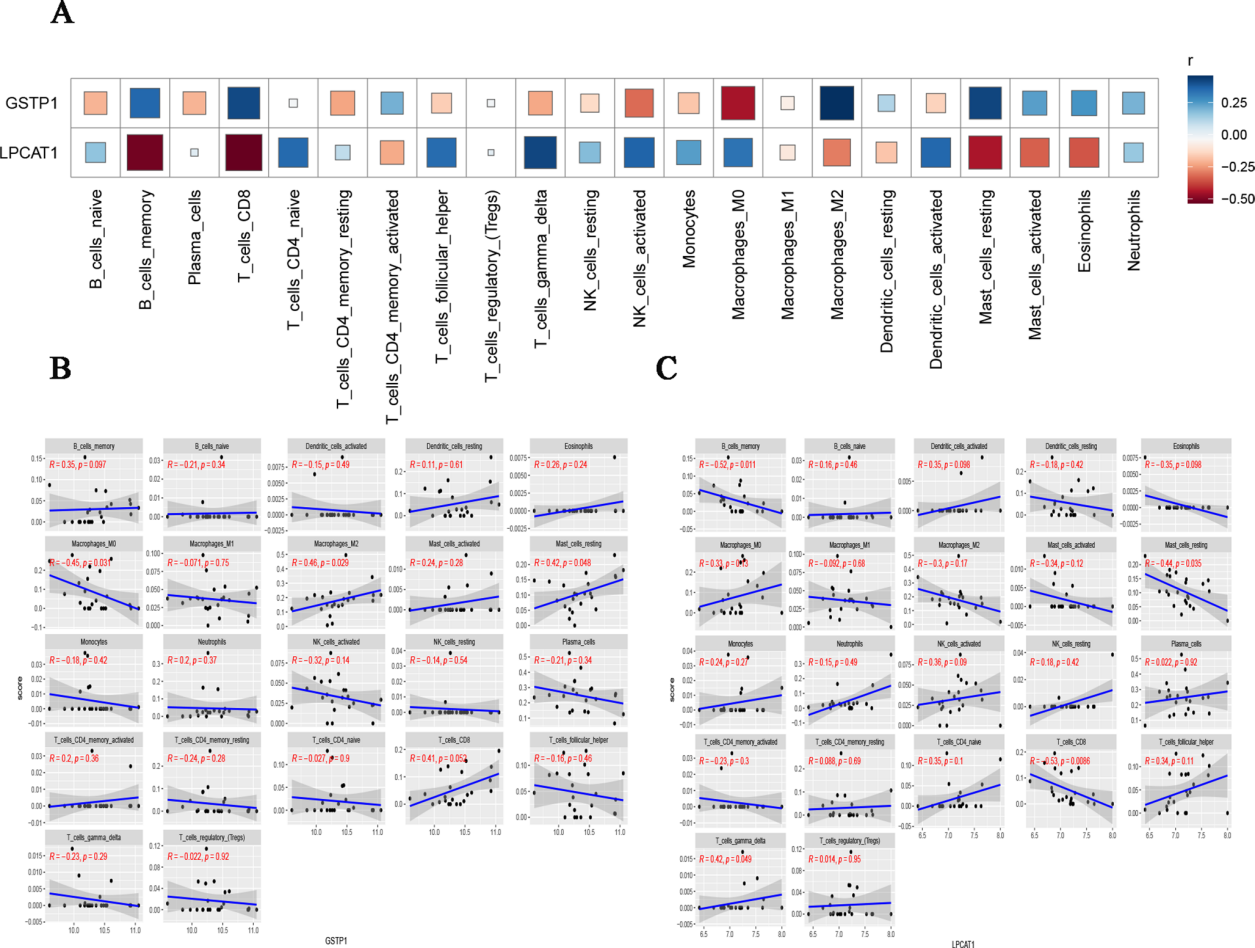
Hub ARGs and immune cell infiltration correlation. (**A**) Heatmap illustrating the correlation coefficients between the expression of hub ARGs (*GSTP1* and *LPCAT1*) and the infiltration levels of 22 immune cell types. Red and blue represent positive and negative correlations, respectively; color intensity indicates the strength of correlation. (**B**) Scatter plots showing Spearman correlation between *GSTP1* expression and immune cell abundance. Each subplot represents a specific immune cell type. Black dots denote individual samples, blue lines show the fitted regression trend, and shaded areas represent the 95% confidence interval. Correlation coefficient (ρ) and *P*-value are shown in red. (**C**) Similar to (B), Spearman correlation between *LPCAT1* expression and immune cell infiltration is shown. Statistically significant correlations (*P* < 0.05) are marked accordingly

We analyzed the influence of the two hub ARGs on immune infiltration and conducted a Spearman correlation analysis on gene expression levels and immune cell contents. To investigate the influence of *GSTP1* and *LPCAT1* on the PCOS immune microenvironment, we correlated their expression with the abundance of 22 immune cell types (Fig. 5A). *GSTP1* expression showed significant negative correlations with M0 macrophages (*r* = -0.45), suggesting a role in suppressing pro-inflammatory immunity (Fig. 5A and B). Consistent with these correlations, analysis of *GSTP1* expression levels (Fig. 5B) revealed increased infiltration of M2 macrophages (*r* = 0.46) increased *GSTP1* expression, along with an increase in resting mast cells (*r* = 0.42).

Conversely, *LPCAT1* expression was strongly positively correlated with T cells of the gamma delta subtype (*r* = 0.42; Fig. 5C), indicating a link to these potentially immunosuppressive cells. *LPCAT1* also showed negative correlations with B cells memory (*r* = -0.52) and T cells CD8 (*r* = -0.53), and in resting mast cells (*r* = -0.44), see detail in Fig. 5C.

### Construction of ceRNAs and Prediction of Small Molecule Drugs

**Fig. 6:**
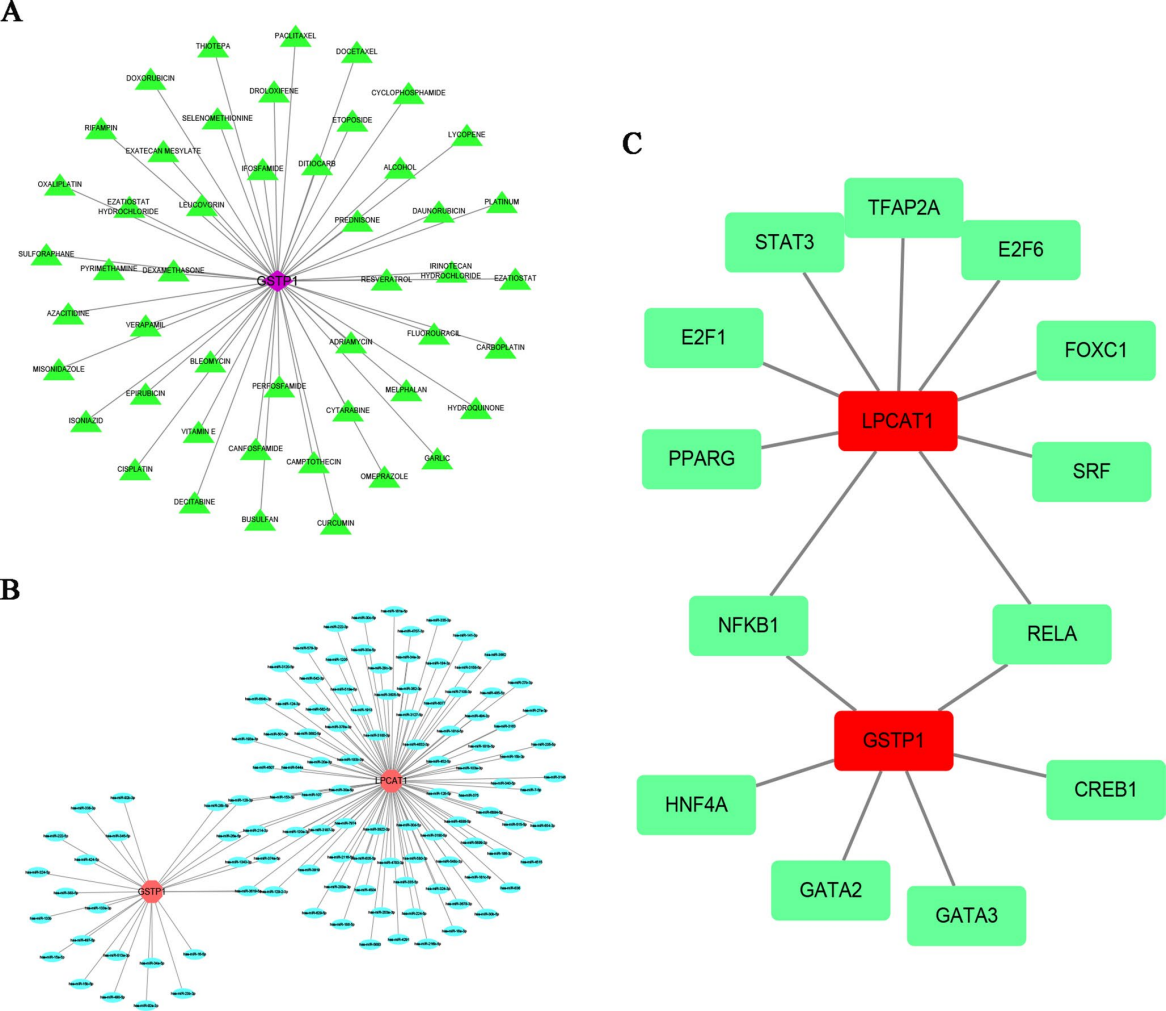
Drug prediction and ceRNA network construction. (**A**) Drug–Gene Interaction Database (DGIdb) was used to predict drugs that interact with *GSTP1*. The network identified 48 drug-gene pairs. Purple shape represents *GSTP1*, while green shapes represent potential drugs. (**B**) Competing endogenous RNA (ceRNA) network based on hub genes *GSTP1* and *LPCAT1*. The network shows predicted miRNA-mRNA interactions in PCOS. Red nodes indicate genes, and blue ellipses indicate miRNAs. (**C**)The transcription factor (TF)-gene co-regulatory network in PCOS interaction of TF with two hub genes. The red shapes represent genes, and the green shapes represent TFs

Using the bioinformatics tool “multiMiR,” key genes were predicted, and a competitive endogenous RNA (ceRNA) network was constructed with miRNA as a mediator to predict potential small molecule drugs. By integrating information from the DGIbd database, further predictions of gene-drug targets were made. Leveraging the NetworkAnalyst database, the interactions between transcription factors and hub ARGs were analyzed, resulting in a TF-target gene regulatory network diagram. *GSTP1* is closely related to 26 miRNAs, including hsa-miR-133a-3p, hsa-miR-345-5p, and hsa-miR-374a-5p. Similarly, *LPCAT1* is associated with 96 miRNAs (as illustrated in Fig. 6C), including hsa-miR-205-5p, hsa-miR-126-5p, and hsa-miR-141-3p. In the transcription factor-target gene regulatory network, we found that transcription factors such as STAT3 and PPARG were closely related to these two hub ARGs (Fig. 6B). Through the DGIbd database, we predicted a total of 49 drugs for the prediction of PCOS with *GSTP1* (Fig. 6A), including VITAMIN E, CURCUMIN, and RESVERATROL, suggesting their possible roles as therapeutic candidates warranting further investigation.

### Pancancer Expression of *GSTP1* and *LPCAT1*

**Fig. 7:**
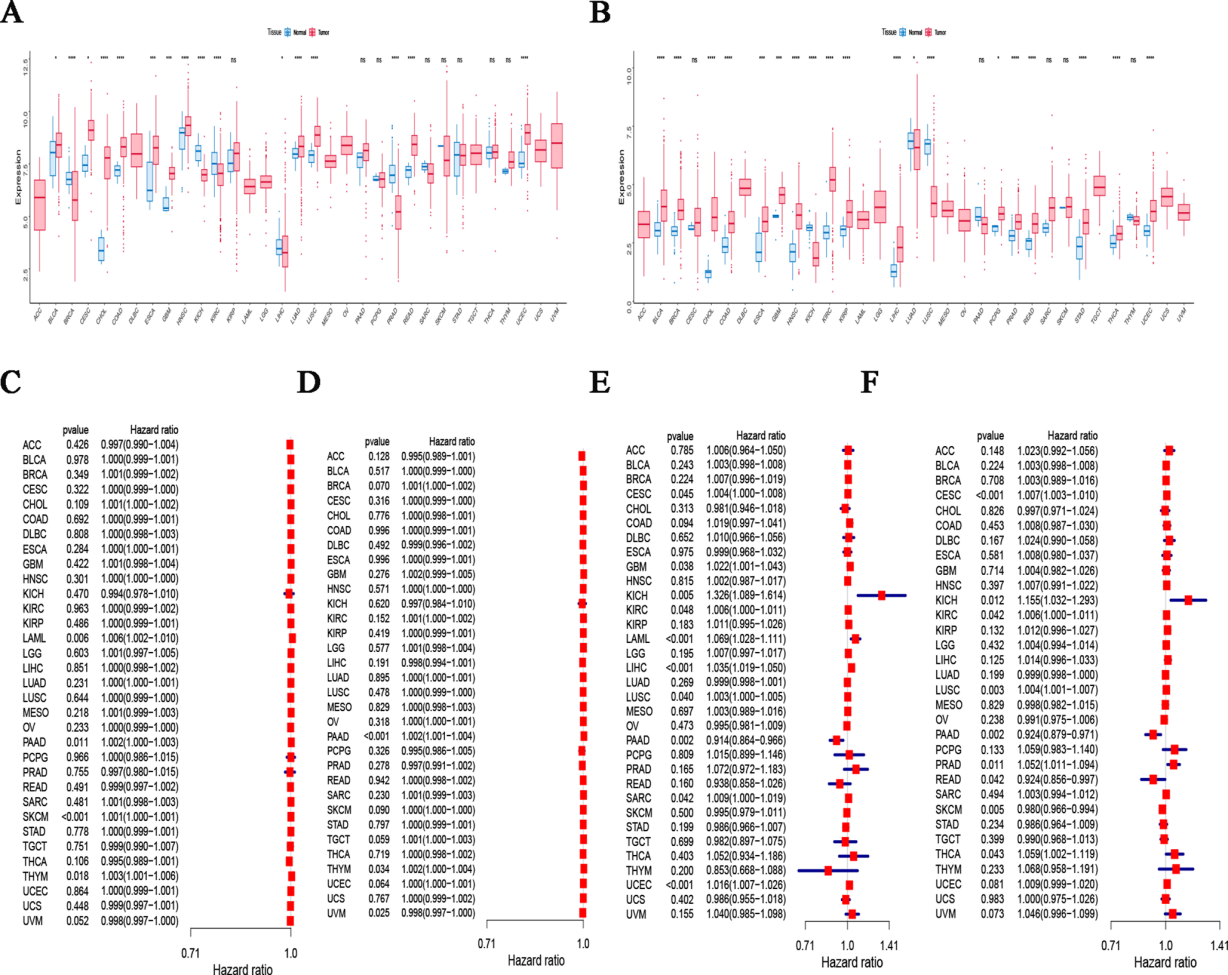
Pan-cancer analysis in PCOS with shared hub genes. (**A**) Analyzing the expression disparities of *GSTP1* across 33 different types of cancers through the TCGA database. (**B**) Analyzing the expression disparities of *LPCAT1* across 33 different types of cancers through the TCGA database. (**C**) A forest plot depicting *GSTP1* hazard ratio along with its 95% confidence interval, encompassing overall survival (OS) rates for 33 distinct cancers were presented. (**D**) A forest plot depicting *GSTP1* hazard ratio along with its 95% confidence interval, encompassing progression-free interval (PFI) rates for 33 distinct cancers were presented. (**E**) A forest plot depicting *LPCAT1* hazard ratio along with its 95% confidence interval, encompassing overall survival (OS) rates for 33 distinct cancers were presented. (**F**) A forest plot depicting *LPCAT1* hazard ratio along with its 95% confidence interval, encompassing progression-free interval (PFI) rates for 33 distinct cancers were presented. Note: The symbol in the figures * represents *P* < 0.05; ** represents *P* < 0.01; *** represents *P* < 0.001; **** represents *P* < 0.0001

In the TCGA dataset, the pancancer expression profiles of *GSTP1* and *LPCAT1* are shown in Fig. 7A and B. We observed increased expression levels of *GSTP1* in LUSC, BLCA, CESC, HNSC, LUAD, LUAD, and READA. However, it exhibited lower expression levels in cancers such as CESC, ESCA, GBM, and PRAD. *LPCAT1* expression was elevated in BLCA, BRCA, CHOL, COAD, ESCA, GBM, HNSC, KIRC, LIHC, PRAD, and STAD tumors. Conversely, it displayed lower expression levels in KICH.

We investigated the correlation between *GSTP1* expression and the prognosis of a wide range of cancer patients, including overall survival (OS) and progression-free interval (PFI) data. According to the OS analysis, *GSTP1* expression was significantly linked to both OS and the PFI in PAAD patients and THYM patients and acted as a risk factor (Fig. 7C and D). *LPCAT1* was a risk factor for CESC, GBM, KICH, KIRC, LAML, LIHC, LUSC, SARC, and UCEC, while exerting protective effects on PAAD in the OS study (Fig. 7E). According to the PFI analysis, *LPCAT1* expression was significantly correlated with five types of cancer, with READ, SKCM, and PAAD being protective factors and with CESC, KICH, KIRC, LUSC, PRAD and THCA being risk factors (Fig. 7F).

### Pancancer Expression and Immune Infiltration

**Fig. 8:**
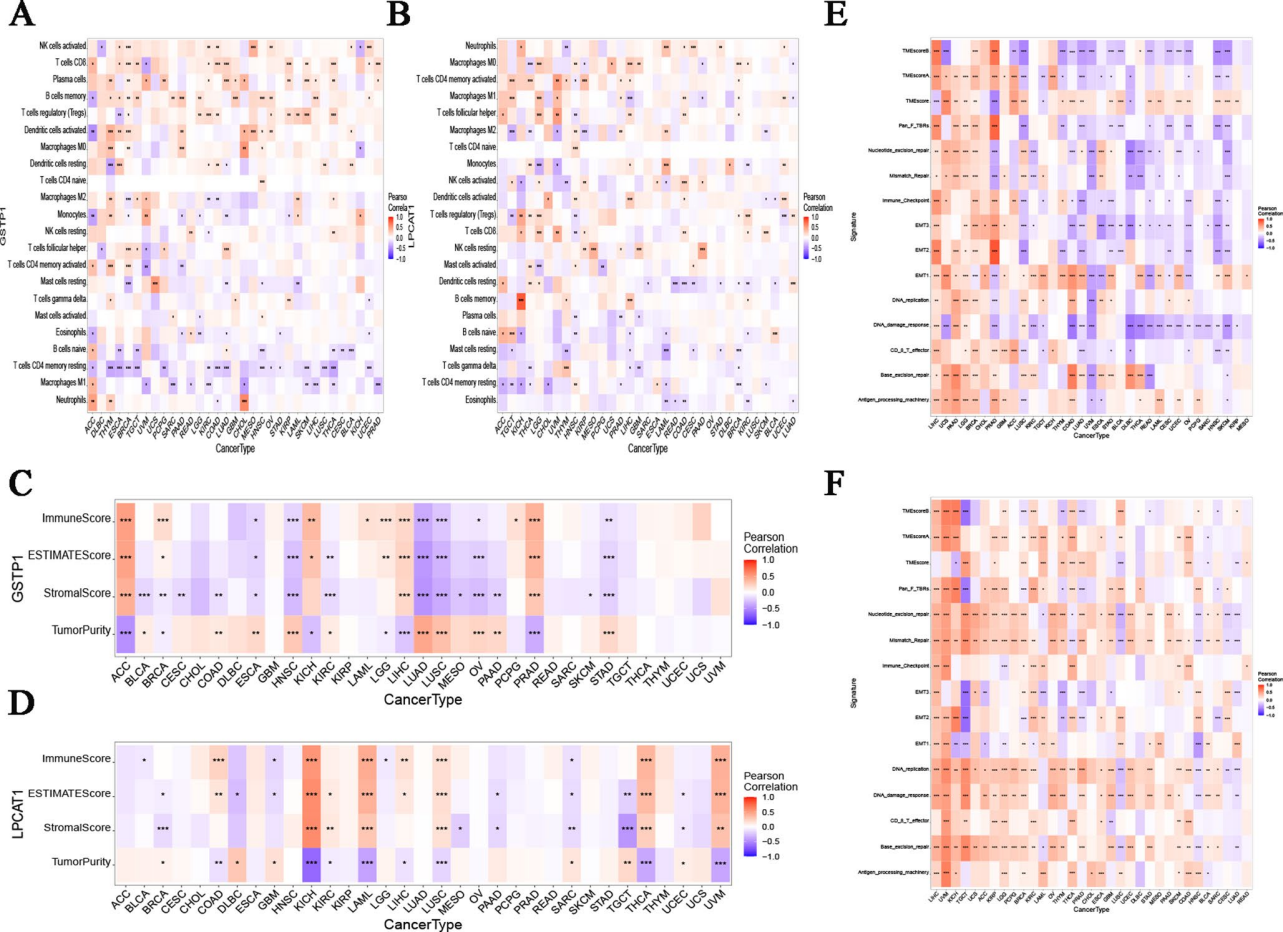
Hub genes and their correlations with pan-cancer and immune cells. (**A**) Hierarchical clustering of the distribution of *GSTP1* and the 22 immune cells with 33 different types of cancers. Colors indicate correlation strength (red = positive, blue = negative). (**B**) Hierarchical clustering of the distribution of *LPCAT1* and the 22 immune cells with 33 different types of cancers. (**C**) Correlation analysis of immune cell infiltration immunophenoscores, stromal Scores, and estimation scores with *GSTP1* across 33 different types of cancers samples. (**D**) Correlation analysis of immune cell infiltration immunophenoscores, stromal Scores, and estimation scores with *LPCAT1* across 33 different types of cancers samples. (**E**) Hierarchical clustering of the associationssociation analysis of immune regulatory factors between *GSTP1* expression and 33 different types of cancers samples. (**F**) Hierarchical clustering of the association analysis of immune regulatory factors between *LPCAT1* expression and 33 different types of cancers samples. Note: The symbol in the figures * represents *P* < 0.05; ** represents *P* < 0.01; *** represents *P* < 0.001; **** represents *P* < 0.0001

The tumor microenvironment is primarily composed of tumor-associated fibroblasts, immune cells, extracellular matrix, various growth factors, inflammatory factors, and specific physicochemical characteristics, along with the cancer cells themselves. The tumor microenvironment significantly influences tumor or nontumor immune-mediated diagnosis, survival outcomes, and clinical treatment sensitivity. Given the pivotal role of immune responses not only in PCOS but also in cancer pathogenesis, we employed *GSTP1* and *LPCAT1* to investigate potential associations between these two disease categories. The CIBERSORT results indicated a significant correlation between the levels of immune cell infiltration and *GSTP1* expression across most types of cancers (Fig. 8A). The graph reveals a positive correlation among neutrophils, resting mast cells, and activated dendritic cells. *LPCAT1* was significantly positively correlated with B-cell memory, as well as with resting NK cells (Fig. 8B). The ESTIMATE algorithm was used to calculate the estimated scores for the stromal and immune components. Our findings related to immune infiltration demonstrated a strong association between *GSTP1* expression and ACC, HNSC, LUAD, LUSC, and PRAD, whereas *LPCAT1* expression was linked to KICH, LAML, and THCA (Fig. 8C and D). Furthermore, gene coexpression analysis was conducted to explore the relationships between *GSTP1* and *LPCAT1* expression and the tumor microenvironment (TME) and between GSTP1 and 33 immune-related genes in tumors in the TCGA database. Additionally, significant correlations were detected between *GSTP1*, *LPCAT1*, and commonly related regulatory genes, such as immune checkpoint-related genes and CD8 T effector genes (Fig. 8E and F).

### MGI-Based Functional Annotation

In the MGI database, both *GSPT1* and *LPCAT1* exhibit positive phenotypes potentially related to the underlying mechanisms of PCOS. *GSPT1* was implicated in the reproductive system, endocrine glands, metabolic homeostasis, adipose tissue, and neurobehavioral functions, suggesting its involvement in ovarian function regulation, hormone metabolism, insulin resistance, and physiological processes linked to PCOS-related obesity and mood disorders. *LPCAT1* also shown phenotypes associated with the reproductive system, endocrine regulation, metabolic functions, and adipose tissue, indicating a potential role in hormone synthesis, energy metabolism, and the manifestation of PCOS phenotypes. See Supplementary Tables 1 and 2 for details.

## Discussion

Anoikis, a vital defense mechanism in organisms, prevents detached cells from mislocating and disrupting normal growth. Various triggers, including DNA damage and endoplasmic reticulum stress, activate anoikis, primarily regulated by mitochondria [42]. Disrupted apoptosis is observed in diseases like cardiovascular ailments, diabetes, and reproductive disorders [43–45]. In polycystic ovary syndrome (PCOS), characterized by hormonal imbalance and ovarian cysts, compromised cell-matrix interactions may induce anoikis [46]. Decreased extracellular matrix (ECM) in PCOS ovaries hampers cell survival, generating reactive oxygen species (ROS) and influencing apoptosis [47–49]. Understanding anoikis in PCOS elucidates its pathogenesis. Mining the genomic features associated with ankinois-PCOS contributes to understanding, diagnosing, and individualizing the treatment of PCOS, while laying the groundwork for elucidating its immune mechanisms and developing immune-modulating therapies.

In this research, we combined diverse bioinformatics techniques, such as WGCNA, machine learning, immune cell analysis, and pancancer studies, to investigate how ARGs influence PCOS. The immue infiltration analysis between healthy controls and PCOS cases suggested that both innate and adaptive immune components are suppressed in the PCOS immune microenvironment. The reduction in CD8⁺ T cells, key effectors in cytotoxic immune defense, may reflect impaired adaptive immune activation, consistent with previous reports showing diminished T cell cytotoxicity in PCOS [50]. The decreased abundance of memory B cells and plasma cells further points to weakened humoral immunity [51]. Meanwhile, the increased proportions of M2 macrophages and Tregs were in line with prior studies indicating an anti-inflammatory shift or immune tolerance mechanism in PCOS, possibly contributing to chronic low-grade inflammation and ovarian dysfunction [52].

We further analyzed gene expression in healthy and PCOS samples, revealing differences. Through WGCNA, we identified 13 dysfunctional hub ARGs in PCOS patients, suggesting a potential role for anoikis in PCOS development. This insight provides strong support for understanding PCOS pathology. We then used LASSO and random forest algorithms and identified *GSTP1* and *LPCAT1* as hub genes related to PCOS. The ROC analysis confirmed that these genes exhibited robust model prediction accuracy (AUC > 0.80), as confirmed by nomogram analysis and Mouse Genome Informatics (MGI). *GSPT1* (G1 to S phase transition 1) functions as a translation termination factor critical for cell cycle regulation and is vital for cell cycle regulation [53], while *LPCAT1* is key for lipid remodeling and is associated with cancers and nontumor diseases [54, 55]. *LPCAT1* expression also increases in early pregnancy trophoblast cultures [56]. Although there is currently no literature confirming the relationship between these two genes and PCOS, it will be necessary for future research to conduct more in-depth experimental studies.

Through GO and KEGG analyses, the two hub ARGs were found to potentially be involved in signaling pathways associated with PI3K, atherosclerosis, apoptosis, and immune-related pathways in PCOS patients. Immune cell infiltration analysis with the two target ARGs indicated that *GSTP1* expression was positively correlated with immune-infiltrating cells such as M0 macrophages, while *LPCAT1* expression was positively correlated with γδ T cells and negatively correlated with memory B cells and CD8 + T cells. These findings are consistent with previous research indicating immune cell and cytokine imbalances in the early stages of PCOS [57]. The reduction in CD8⁺ T cells, key effectors in cytotoxic immune defense, may reflect impaired adaptive immune activation, consistent with previous reports showing diminished T cell cytotoxicity in PCOS [58]. The decreased abundance of memory B cells and plasma cells further points to weakened humoral immunity [59]. Meanwhile, the increased proportions of M2 macrophages and Tregs were in line with prior studies indicating an anti-inflammatory shift or immune tolerance mechanism in PCOS, possibly contributing to chronic low-grade inflammation and ovarian dysfunction [53]. We investigated these two hub genes by analyzing distinct ceRNAs and miRNAs in the transcription factor–target gene network, revealing potential interacting ceRNAs. We found that several vital miRNAs involved in PCOS, such as hsa-miR-205-5p and hsa-miR-345-5p, are associated with ARGs-PCOS. The relevant transcription factors include STAT3. We collected and curated information regarding the interactions between drugs and the two hub genes using DGIdb. Curcumin, resveratrol, and vitamin E were identified as potential therapeutic agents that act on PCOS mechanisms by targeting *GSTP1*. Insulin resistance (IR) is closely associated with PCOS and is one of the most prevalent endocrine disturbances in 75% of PCOS patients; IR is linked to PI3K and apoptosis signaling pathways [60, 61], and STAT3 may influence the metabolism of PCOS patients by impacting insulin signaling pathways and metabolic regulation. Additionally, STAT3 may also regulate inflammation and immune responses, leading to chronic inflammation and immune system abnormalities in PCOS patients [62]. Macrophages and dendritic cells, along with other immune cells, participate in the occurrence and progression of chronic inflammation in PCOS patients. Macrophages are essential cells in the immune response and play a particularly significant role in PCOS [63]. Reduced intrafollicular dendritic cells and disrupted cytokine balance in PCOS patients might impact follicular development. The dysregulation of genes associated with immune modulation may serve as molecular clues to the aberrant follicular development and anovulation observed in patients with PCOS [64]. Previous studies have indicated that in PCOS, the expression of hsa-miR-205-5p and hsa-miR-34a-5p is upregulated [65, 66]. In conjunction with the findings of this study, we hypothesized that these microRNAs may be involved in follicle development and oocyte maturation by targeting hub ARGs. Further analysis of the Mouse Genome Informatics (MGI) database also revealed that *GSPT1* and *LPCAT1* played critical roles in embryonic development, metabolic regulation, reproductive function, and disease pathogenesis in mouse models. *GSPT1* was implicated in the development of metabolic disorders through its effects on the cell cycle and protein synthesis, while *LPCAT1* influenced lung function and reproductive health by regulating the phospholipid composition of cell membranes. Furthermore, aberrant expression of *LPCAT1* was closely associated with the progression of various cancers. Given the close link between metabolic dysregulation, reproductive dysfunction, and the pathophysiology of PCOS [1], these findings suggest that *GSPT1* and *LPCAT1* may also contribute to the onset or progression of PCOS, warranting further investigation in human reproductive and metabolic contexts. To strengthen this hypothesis, we further examined phenotypic annotations in the MGI database and found that mouse models with altered expression of *GSPT1* or *LPCAT1* exhibit features such as abnormal ovarian morphology, impaired folliculogenesis, hormonal imbalances (e.g., altered estrogen and gonadotropin levels), and metabolic disturbances including insulin resistance and glucose intolerance. These phenotypes closely parallel hallmark manifestations of PCOS in humans—namely ovulatory dysfunction, hyperandrogenism, and metabolic dysregulation—suggesting a conserved functional role of these genes across species.

Drug prediction analysis suggested that compounds such as vitamin E, curcumin, and resveratrol may have therapeutic potential by targeting anoikis-related pathways associated with PCOS. Researchers have shown that curcumin treatment can protect the ovaries, reduce testosterone levels, restore IR, decrease the infiltration of inflammatory cells, and modulate signaling pathways [67]. Curcumin may promote ovarian morphological changes by regulating the IRS1/PI3K/GLUT4 pathway [68]. Resveratrol might have a potential role in regulating androgen levels, improving insulin sensitivity, and mitigating inflammation, among other effects, which could play a certain role in PCOS. Its potential actions in terms of antioxidation, anti-inflammation, and hormone regulation have drawn significant attention in relation to PCOS [69, 70]. Vitamin E, a crucial fat-soluble vitamin, may be involved in the treatment of PCOS by participating in antioxidative processes, hormone level equilibrium, and IR enhancement [71]. It is important to note that our drug prediction was based on bioinformatics inference and should be considered hypothesis-generating until validated by functional studies, and further pharmacological and functional studies are needed to evaluate their efficacy and mechanisms in the context of PCOS. In the pancancer analysis, we revealed a close association between these two ARGs and various types of tumors. Furthermore, we showed that these two ARGs are closely correlated with immune cell infiltration and immune checkpoint genes in the TME.

Our research has several limitations. First, despite the use of data from public databases, the sample size of PCOS patients was relatively small (*n* = 23), which may limit the statistical power and generalizability of our conclusions. Relatedly, we must recognize that PCOS prevalence, phenotypes, and associated immune features can vary significantly across different geographical locations and ethnicities1 [72]. This necessitates future research to consider these factors when investigating the role of *GSTP1* and *LPCAT1* in PCOS-related immune dysregulation, ensuring the generalizability of findings across diverse populations. Second, although the study lacked direct biological validation in patient-derived tissues or cell lines, the robustness of our predictive model was supported by comprehensive bioinformatics analysis. Future work will involve experimental confirmation through qRT-PCR and Western blotting in granulosa cells (GCs) lines and animal models. Similarly, while CIBERSORT-based immune deconvolution provided valuable insights into immune landscape alterations, these computational findings require validation using techniques such as flow cytometry and immunohistochemistry—particularly regarding M2 macrophage enrichment and CD8⁺ T cell reduction in PCOS ovarian tissues. Third, although MGI database analysis provided functional support, the reliance on computational prediction necessitates further mechanistic studies to clarify how these genes contribute to PCOS pathophysiology. It also remains to be determined whether their expression varies by ethnicity or region, potentially influencing disease heterogeneity. Fourth, the pancancer analysis was intended as an exploratory approach to identify systemic molecular patterns potentially relevant to PCOS. While such cross-disease comparisons offer novel insights, these results should be interpreted with caution and not as direct evidence of PCOS pathophysiology. Moreover, drug-gene interaction analysis identified natural compounds such as resveratrol, curcumin, and vitamin E as potential therapeutics. However, these results are hypothesis-generating and require pharmacological and functional validation to assess therapeutic efficacy and underlying mechanisms. Finally, translation into clinical practice will require standardized biomarker assays, large-scale validation, and integration into diagnostic workflows. Practical challenges such as regulatory approval and cost may also limit implementation of precision medicine in PCOS care.

## Conclusion

This is the first computational study to identify *GSTP1* and *LPCAT1* from RNA-seq data as potential novel prognostic biomarkers for PCOS. Hub ARGs may be implicated in the pathophysiological processes of PCOS through pathways such as PI3K and apoptosis, regulating components such as macrophages and dendritic cells. These findings provide a theoretical foundation for future research into the pathological mechanisms of PCOS, focusing on drug development and the immunomodulatory microenvironment.

## Electronic Supplementary Material

Below is the link to the electronic supplementary material.

Supplementary Material 1

## Data Availability

All data used in this study are available in the GEO repository (https://www.ncbi.nlm.nih.gov/geo/), which including GSE43264 (https://www.ncbi.nlm.nih.gov/geo/query/acc.cgiacc=GSE43264) and GSE98421 (https://www.ncbi.nlm.nih.gov/geo/query/acc.cgiacc=GSE98421). All data generated or analysed during this study are included in this MS. Further inquiries can be directed to the corresponding author.
