# Supplementary material for: Transcriptomic Profiling Reveals Potential Genes Involved in the Immune Landscape of Polycystic Ovary Syndrome: An Exploratory Study
Source: Reprod Sci. 2025 Jun 24;32(7):2404–22. doi: 10.1007/s43032-025-01917-4 (PMC12271263; doi:10.1007/s43032-025-01917-4)
Supplement: Supplementary file 1 — Supplementary Material 1 [file 43032_2025_1917_MOESM1_ESM.doc]

**Figure 1 Study flow**

**Figure 2 Identification of anoikis-related differentially expressed genes (DEGs) and co-expression modules.**

1. Principal component analysis (PCA) showing the sample distribution and batch effect between the two datasets (GSE43264 and GSE98421). Each dot represents a sample; colors indicate dataset origin.
2. Heatmap of differentially expressed genes (DEGs) in polycystic ovary syndrome (PCOS) samples compared to controls. Yellow and blue represent high and low expression levels, respectively.
3. Cluster dendrogram of genes based on weighted gene co-expression network analysis (WGCNA). Each branch represents a gene, and color bars below indicate module membership.
4. Module-trait relationships between gene modules and PCOS/control groups. The color scale reflects correlation coefficients, with red indicating positive and blue indicating negative correlations. Each cell shows the correlation coefficient and p-value in parentheses.
5. (E) Venn diagram showing overlap among DEGs (DIFF), anoikis-related genes (ARGs), and key module genes identified by WGCNA. The intersection of the three sets indicates potential ARGs associated with PCOS.

**Figure 3 Functional enrichment, immune infiltration analysis, and machine learning screening of ARGs in PCOS.**

1. Kyoto Encyclopedia of Genes and Genomes (KEGG) pathway enrichment analysis of PCOS-related ARGs, showing the top enriched signaling pathways.

(B) Gene Ontology (GO) enrichment analysis of PCOS-related ARGs, classified into biological process (BP), cellular component (CC), and molecular function (MF) categories.

(C) Heatmap showing pairwise correlations among immune cell types in PCOS samples. Red and blue colors indicate positive and negative correlations, respectively; numeric values represent correlation coefficients.

(D) Boxplots comparing the relative abundance of immune cell types between PCOS and control groups. Boxes show interquartile ranges, and whiskers indicate data variability. Statistical significance: *P* < 0.05 (*), *P* < 0.01 (**), ns = not significant.

(E) LASSO logistic regression model used for feature selection of ARGs. The plot shows tenfold cross-validation results across different log(λ) values; the vertical dotted line marks the optimal λ with the minimum binomial deviance.

(F) LASSO coefficient profiles of ARGs along the L1 regularization path. Five genes with non-zero coefficients were selected for further analysis.

(G) Venn diagram showing the overlapping ARGs identified by both LASSO and Random Forest algorithms. The intersection indicates the common hub genes selected by both machine learning methods. Percentages represent the proportion of genes in each segment.

1. **Figure 4. Construction and validation of a predictive model based on hub ARGs in PCOS.**

(A) A nomogram model integrating the expression levels of *GSTP1* and *LPCAT1* to predict disease risk in PCOS patients. Points are assigned to each gene and summed to estimate the total risk score.

(B) Receiver operating characteristic (ROC) curves evaluating the diagnostic performance of *GSTP1* (AUC = 0.826) and *LPCAT1* (AUC = 0.803) in distinguishing PCOS from control samples.

(C) Single-sample gene set enrichment analysis (ssGSEA) of *GSTP1*, showing its association with multiple KEGG pathways. The plot ranks samples by expression level and highlights pathway enrichment scores.

(D) ssGSEA results for *LPCAT1*, displaying pathway-level enrichment patterns across samples. Color-coded lines indicate different KEGG functional categories.

**Figure 5 Hub ARGs and immune cell infiltration correlation**

1. Heatmap illustrating the correlation coefficients between the expression of hub ARGs (*GSTP1* and *LPCAT1*) and the infiltration levels of 22 immune cell types. Red and blue represent positive and negative correlations, respectively; color intensity indicates the strength of correlation.
2. Scatter plots showing Spearman correlation between *GSTP1* expression and immune cell abundance. Each subplot represents a specific immune cell type. Black dots denote individual samples, blue lines show the fitted regression trend, and shaded areas represent the 95% confidence interval. Correlation coefficient (ρ) and *P*-value are shown in red.
3. (C) Similar to (B), Spearman correlation between *LPCAT1* expression and immune cell infiltration is shown. Statistically significant correlations (*P* < 0.05) are marked accordingly.

**Figure 6 Drug prediction and ceRNA network construction**

1. Drug–Gene Interaction Database (DGIdb) was used to predict drugs that interact with *GSTP1*. The network identified 48 drug-gene pairs. Purple shape represents *GSTP1*, while green shapes represent potential drugs.
2. Competing endogenous RNA (ceRNA) network based on hub genes *GSTP1* and *LPCAT1*. The network shows predicted miRNA-mRNA interactions in PCOS. Red nodes indicate genes, and blue ellipses indicate miRNAs.
3. The transcription factor (TF)-gene co-regulatory network in PCOS interaction of TF with two hub genes. The red shapes represent genes, and the green shapes represent TFs.

**Figure 7 Pan-cancer analysis in PCOS with shared hub genes.**

1. Analyzing the expression disparities of *GSTP1* across 33 different types of cancers through the TCGA database.
2. Analyzing the expression disparities of *LPCAT1* across 33 different types of cancers through the TCGA database.
3. A forest plot depicting *GSTP1* hazard ratio along with its 95% confidence interval, encompassing overall survival (OS) rates for 33 distinct cancers were presented.
4. A forest plot depicting *GSTP1* hazard ratio along with its 95% confidence interval, encompassing progression-free interval (PFI) rates for 33 distinct cancers were presented.
5. A forest plot depicting *LPCAT1* hazard ratio along with its 95% confidence interval, encompassing overall survival (OS) rates for 33 distinct cancers were presented.
6. A forest plot depicting *LPCAT1* hazard ratio along with its 95% confidence interval, encompassing progression-free interval (PFI) rates for 33 distinct cancers were presented.

**Figure 8 Hub genes and their correlations with pan-cancer and immune cells**

1. Hierarchical clustering of the distribution of *GSTP1* and the 22 immune cells with 33 different types of cancers. Colors indicate correlation strength (red = positive, blue = negative).
2. Hierarchical clustering of the distribution of *LPCAT1* and the 22 immune cells with 33 different types of cancers.
3. Correlation analysis of immune cell infiltration immunophenoscores, stromal Scores, and estimation scores with *GSTP1* across 33 different types of cancers samples.
4. Correlation analysis of immune cell infiltration immunophenoscores, stromal Scores, and estimation scores with *LPCAT1* across 33 different types of cancers samples.
5. Hierarchical clustering of the associationssociation analysis of immune regulatory factors between *GSTP1* expression and 33 different types of cancers samples.
6. Hierarchical clustering of the association analysis of immune regulatory factors between *LPCAT1* expression and 33 different types of cancers samples.

Note: The symbol in the figures * represents *P* < 0.05; ** represents *P* < 0.01; *** represents *P* < 0.001; **** represents *P* < 0.0001

Abbreviations: GEO (Gene Expression Omnibus), PCOS (Polycystic Ovary Syndrome), DEGs (Differential Expression Genes), WGCNA (Weighted Gene Co-expression Network Analysis), ARGs (Anoikis-Related Genes), MGI (Mouse Genome Informatics) database, GO (Gene Ontology), KEGG (Kyoto Encyclopedia of Genes and Genomes), ROC (Receiver Operating Characteristic), GSEA (Gene Set Enrichment Analysis), LASSO (Least Absolute Shrinkage and Selection Operator), TCGA (The Cancer Genome Atlas); TME (Tumor Microenvironment)
